# Supplementary material for: The functional logic of odor information processing in the Drosophila antennal lobe
Source: PLoS Comput Biol. 2023 Apr 21;19(4):e1011043. doi: 10.1371/journal.pcbi.1011043 (PMC10156017; doi:10.1371/journal.pcbi.1011043)
Supplement: S1 Appendix — S1 Table. Glossary of terms and abbreviations. S2 Table. Glossary of mathematical notation. S3 Table. Model description of the single-channel AL circuit in S2 Fig in S1 Appendix. Note that for notational simplicity the parameter values for θ are omitted. Indices under the Model column refer to the following detailed models: (1) OSN to PreLN Synapse, (2) Pre-LN BSG, (3) OSN Axon-Terminal. The Pre-LN pathway receives as input the neurotransmitter concentration of OSN Axon-Terminal in the same channel and outputs a single Pre-LN spike train. Note that only components in the DNP-FB [I] pathway are specified as the other circuit components are the same as in Fig 10(Left) and are specified in Table 1. The single-channel Pre-LN pathway is modeled as a DNP-FB[I]. S4 Table. Model description of the multi-channel AL circuit in S3 Fig in S1 Appendix. Note that for notational simplicity the parameter values for θ are omitted. Indices under the Model column refer to the following detailed models: (1) OSN to PreLN Synapse in the r-th channel, (2) Pre-LN BSG, (3) OSN Axon-Terminal in the r-th channel. Note that only components in the Global DNP-FB pathway are specified as the other circuit components are the same as in Fig 11(Left) for each of the R channels. The differential equations for each model are detailed under the Equation column, with the corresponding parameters summarized under the Parameters column. Note that under the Parameters column, values for the fixed parameters are shown. The circuit has a total of 23 free parameters, the same as the single-channel AL circuit in S2 Fig in S1 Appendix. and S3 Table in S1 Appendix. The correspondence between the Pre-LN pathway and the Global DNP-FB model is made explicit on right side of rows (1–3). S1 Fig. Concentration Invariance and ON/OFF Contrast Boosting of OR59b OSN and DM4 PN I/O pairs. (A[i]) Acetone odorant concentration waveforms. (A[ii]) Acetone odorant concentration contrast computed with ε = 1. (A[iii]) Or59b OSN P [file pcbi.1011043.s001.pdf]

The Functional Logic of Odor Information Processing in the *Drosophila* Antennal Lobe: Supporting Information

S1 Table in S1 Appendix. Glossary of terms and abbreviations.

Acronym/Notation	Full Term	Definition
BSG	Biophysical Spike Generator	Models the spike generation processes of neurons.
CSN	Connor-Stevens Neuron	Point neuron model for all BSGs in the AL circuit.
Phase Space		A space representing all states of a dynamical system (e.g., BSG).
Stable Attractors		A set of stable limit cycles in the phase-space of PN BSGs.
AL	Antennal Lobe	Neuropil in the *Drosophila* early olfactory system.
OSN	Olfactory Sensory Neuron	Sensory neurons in the Antenna.
PN	Projection Neuron	Output Neurons of the AL. In the current work, PN refers to an uni-glomerular Project Neuron.
Pre-LN	Presynaptic Local Neuron	Pan-glomerular inhibitory Local Neuron providing presynaptic inhibition to an OSN Axon-Terminal. Represented as superscript L.
Post-eLN	Postsynaptic Excitatory Local Neuron	Uni-glomerular excitatory Local Neuron providing postsynaptic excitation to a PN. Represented as superscript eL.
Post-iLN	Postsynaptic Inhibitory Local Neuron	Uni-glomerular inhibitory Local Neuron providing postsynaptic inhibition to a PN. Represented as superscript iL.
Differential DNP	Differential Divisive Normalization Processor	A class of dynamic circuit models advanced in the current work (**Eq.**(1) , **Eq.**(3)).
DNP-FF	Feedforward DNP	DNP model where the inhibitory signal is the processed feedforward input signal (**Eq.**(3)).
DNP-FB	Feedback DNP	DNP model where the inhibitory signal is the processed feedback output signal (**Eq.**(1)).
Global DNP-FB	Spatio-temporal DNP-FB	Multi-channel feedback DNP model where the inhibitory signal integrates all feedback output signals (**Eq.**(4)).
Global DNP-FF	Spatio-temporal DNP-FF	Multi-channel feedforward DNP model where the inhibitory signal integrates all feedforward input signals (**Eq.**(4)).
———	Channel	A sub-circuit formed by OSNs expressing a given receptor type, its downstream PNs and the glomerulus connecting the OSNs and PNs.
PSTH	Peri-Stimulus Time Histogram	An estimate of BSG spike rate from spike timing. Also referred to as spike rate.
Concentration Invariance		The feature characterizing a component of the PN response that is invariant to concentration amplitude (see **Fig 2**).
Concentration Contrast		A temporal signal computed from the concentration waveform of odor input. See **Section 4.2** for detailed definition.
Contrast Boosting		The feature characterizing a component of the PN response that is proportional to concentration contrast (see **Fig 2**).
Semantic Information		The identity of an odorant (e.g., Acetone) that is represented at the PN level as a set of stable attractors (see next entry).
Syntactic Information		The Shannon information of odorant concentration waveforms.
Semantic Timing		Onset/offset timing events indicating the presence/absence of an odorant object.
Single-Channel AL Circuit		AL circuit modeling a single glomerulus.
Multi-Channel AL Circuit		AL circuit modeling all glomeruli with known affinity values [1,2].

S1 Fig in S1 Appendix. Concentration Invariance and ON/OFF Contrast Boosting of OR59b OSN and DM4 PN I/O pairs. (A[i]) Acetone odorant concentration waveforms. (A[ii]) Acetone odorant concentration contrast computed with $\varepsilon = 1$. (A[iii]) Or59b OSN PSTHs. (A[iv]) Steady-state components of Or59b OSN PSTHs. (A[v]) Transient components of Or59b OSN PSTHs. (B[i]) Acetone odorant concentration waveforms. (B[ii]) Acetone odorant concentration contrast computed with $\varepsilon = 1$. (B[iii]) DM4 PN PSTHs. (B[iv]) Steady-state components of DM4 PN PSTHs. (B[v]) Transient components of DM4 PN PSTHs. (C[i]) Maximum cross-correlations between the odorant concentration waveforms and Or59b OSN and DM4 PN PSTHs. Maximum cross-correlation is defined as $\max_\tau \int_0^T u(t+\tau)v(t)dt$, where $u(t)$ is the odorant concentration waveform in (A[i]/B[i]) and $v(t)$ is the Or59b OSN/DM4 PN PSTH in (A[iii]/B[iii]), with $T = 17.5$ seconds (duration of the signals $u(t), v(t)$) and $\tau \in [-500, 500]$ milliseconds. Each black dot in (C[i]) corresponds to a maximum cross-correlation of a single experiment in (A)/(B). (C[ii]) Same as (C[i]) but for the odorant concentration waveforms and steady-state components of Or59b OSN/DM4 PN PSTHs. (C[iii]) Same as (C[i]) but for the transient components of the odorant concentration contrast and Or59b OSN/DM4 PN PSTHs. Note that, (C[i, ii]) indicate that both the overall PSTH and the steady-state component of the DM4 PN PSTH response are less correlated with the concentration waveform than the Or59b OSN PSTH response is correlated with the concentration waveform, suggesting concentration-invariance at the PN level. (C[iii]) indicates that the transient component of DM4 PN response is more correlated with the concentration contrast than the Or59b OSN response is correlated with the concentration contrast, suggesting contrast-boosting at the PN level.

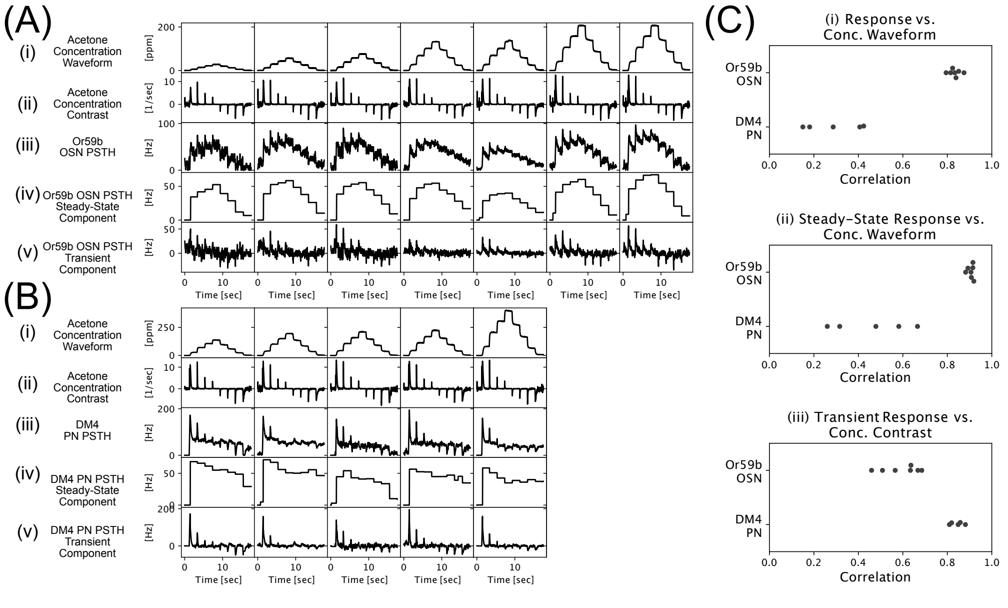

S2 Table in S1 Appendix. Glossary of mathematical notation.

Notation	Definition
$\boldsymbol{\lambda}(t;\boldsymbol{\theta})$	The BSG PSTH of the model spike train.
$\hat{\boldsymbol{\lambda}}(t)$	The BSG PSTH of the physiological recording data.
$\sum_k \delta(t-t_k;\boldsymbol{\theta})$	Spike train of a BSG model represented as a sum of delta pulses.
$\boldsymbol{\theta}=(\boldsymbol{\theta}_1,\boldsymbol{\theta}_2,\boldsymbol{\theta}_3)$	A vector of free parameter values for circuit components in the AL models.
$\boldsymbol{\theta}_1$	A vector of free parameters for circuit components in the Pre-LN pathway (e.g., see Table 1. models (1,3), Table 2. models (1,3)). For model described in Table 1., $\boldsymbol{\theta}_1=(\alpha_1,\beta_1,\overline{g}^{OL}_{max},\alpha_2,\beta_2,\kappa_2,\overline{[NT]}_{max})$.
$\boldsymbol{\theta}_2$	A vector of free parameters for circuit components in the Post-eLN pathway (i.e., see Table 1. model (4)), $\boldsymbol{\theta}_2=(\alpha_3,\beta_3,\kappa_3,\alpha_4,\beta_4,\alpha_5,\beta_5,\overline{g}^{OeL}_{max})$.
$\boldsymbol{\theta}_3$	A vector of free parameters for circuit components in the Post-iLN pathway (i.e., see Table 1. model (6)), $\boldsymbol{\theta}_3=(\alpha_6,\beta_6,\kappa_6,\alpha_7,\beta_7,\alpha_8,\beta_8,\overline{g}^{OiL}_{max})$.
$[\mathbf{b}]_{ro},\mathbf{b}_o$	Rate of binding between odorant o and receptor type r.
$[\mathbf{d}]_{ro},\mathbf{d}_o$	Rate of dissociation of odorant o from a bound receptor r.
$[\mathbf{b}]_{ro}/[\mathbf{d}]_{ro},\mathbf{b}_o\oslash\mathbf{d}_o$	Ratio of binding and dissociation rates.
$[NT](t),\overline{[NT]}_{max}$	Time-varying neurotransmitter concentration and the maximum neurotransmitter concentration level of synapse models. For example, the neurotransmitter concentration released by the OS Axon-Terminal into the synaptic cleft between OSN and PN in **Table 1** Model (3).
V	Membrane voltage of BSG (e.g., Pre-LN membrane voltage in **Table 1** Model (1)).
E	Reversal potential of biophysical synapse model (e.g., **Table 1** Model (8)).
$\sigma^O,\sigma^L,\sigma^{iL},\sigma^{eL},\sigma^P$	Standard deviation of noise injected into the CS point neuron model (e.g., **Table 1** Model (2)). The superscripts O,L,iL,eL,P, respectively denote the BSG models of OSN, Pre-LN, Post-iLN, Post-eLN, and PN.
I^{OP},I^{eLP},I^{iLP}	Synaptic current between OSN Axon-Terminal-to-PN (OP), Post-eLN-to-PN (eLP) and Post-iLN-to-PN (iLP). See **Table 1** Model (8,9,10) for example.
I^{OL},I^{OeL},I^{OiL}	Synaptic current between OSN-to-Pre-LN (OL), OSN-to-Post-eLN (OeL) and OSN-to-Post-iLN (OiL). See **Table 1** Model (1,4,6) for example.
$\mathcal{T}_1,\mathcal{T}_2,\mathcal{T}_3,\mathcal{T}_4$	Nonlinear operators describing dynamics of the single-channel DNP-FF and DNP-FB models.
$\mathcal{T}_5,\mathcal{T}_6,\mathcal{T}_7,\mathcal{T}_8$	Nonlinear operators describing the dynamics of the Global multi-channel DNP-FF and DNP-FB models.
α,β,κ	Parameters of the differential DNP models defined in Eqs. (1,3,4). α amplifies input signal, β describes decay rate of DNP in the absence of input, κ amplifies inhibitory signal in DNP (e.g., **Table 1** Model (3)). These three parameters appear (differentiated by subscripts $1\sim 17$) in all differential DNP model specifications in **Table 1**, **Table 2**, **S3 Table in S1 Appendix** and **S4 Table in S1 Appendix**.
x^{OL},x^{OeL},x^{OiL}	Normalized gating variable (bounded between 0 and 1) of synapse models. Superscripts indicate OL (OSN-to-PreLN), OeL (OSN-to-Post-eLN),OiL (OSN-to-Post-iLN) synapses.
x^{OP},x^{eLP},x^{iLP}	Normalized gating variable (bounded between 0 and 1) of synapse models. Superscripts indicate OP (OSN Axon-Terminal-to-PN), eLP (Post-eLN-to-PN), iLP (Post-iLN-to-PN) synapses.
x_2^{OeL},x_2^{OiL}	Feedforward excitatory internal state variable of OSN-to-PosteLN and OSN-to-PostiLN synapses respectively.
x_3^{OeL},x_3^{OiL}	Feedforward inhibitory internal state variable of OSN-to-PosteLN and OSN-to-PostiLN synapses respectively.
$\overline{g}^{OL}_{max},\overline{g}^{OeL}_{max},\overline{g}^{OiL}_{max}$	Maximum conductances of synapse models. Superscripts indicate OL (OSN-to-PreLN), OeL (OSN-to-Post-eLN),OiL (OSN-to-Post-iLN) synapses.
$\overline{g}^{OP}_{max},\overline{g}^{eLP}_{max},\overline{g}^{iLP}_{max}$	Maximum conductances of synapse models. Superscripts indicate OP (OSN Axon-Terminal-to-PN), eLP (Post-eLN-to-PN), iLP (Post-iLN-to-PN) synapses.

S2 Fig in S1 Appendix. Schematics of the single-channel AL circuit with Pre-LN feedback inhibition and the associated DNP circuit. (Left) Example single-channel AL circuit with Pre-LN feedback inhibition and (Right) the associated DNP circuit. Refer to **S3 Table in S1 Appendix** for model details.

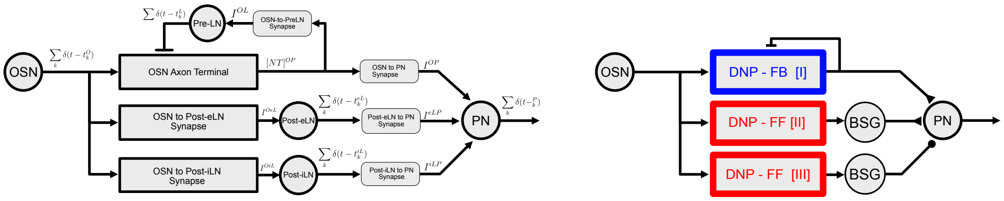

S3 Table in S1 Appendix. Model description of the single-channel AL circuit in S2 Fig in S1 Appendix Note that for notational simplicity the parameter values for $\boldsymbol{\theta}$ are omitted. Indices under the *Model* column refer to the following detailed models: (1) OSN to PreLN Synapse, (2) Pre-LN BSG, (3) OSN Axon-Terminal. The Pre-LN pathway receives as input the neurotransmitter concentration of OSN Axon-Terminal in the same channel and outputs a single Pre-LN spike train. Note that only components in the DNP-FB [I] pathway are specified as the other circuit components are the same as in **Fig 10** (Left) and are specified in **Table 1** . The single-channel Pre-LN pathway is modeled as a DNP-FB[I].

Model	*Parameter*	*Equation*	*Differential DNP Operators*	
(1)	$[\alpha_{12}, \beta_{12}, \bar{g}^{OL}_{max}]$	$\frac{d}{dt}x^{OL} = \alpha_{12} \cdot [NT]^{OP} \cdot (1 - x^{OL}) - \beta_{12} \cdot x^{OL}$<br>$I^{OL} = \bar{g}^{OL}_{max} \cdot x^{OL} \cdot (V^L - E^{OL})$	$\mathcal{T}_1 : \sum_k \delta(t - t^O_{kr}) \mapsto \sum_k \delta(t - t^O_{kr})$<br>$\mathcal{T}_2 : [NT]^{OP} \mapsto \sum_k \delta(t - t^L_k)$	DNP-FB [I]
(2)	$\sigma^L = 0$	$\sum_k \delta(t - t^L_k) \leftarrow \text{NoisyConnorStevens}(I^{OL}; \sigma^L)$		
(3)	$[\alpha_{13}, \beta_{13}, \kappa_{13}, \overline{[NT]}_{max}]$	$\frac{d}{dt}x^{AxT} = \alpha_{13} \cdot \sum_k \delta(t - t^O_k) \cdot (1 - x^{AxT}) - \beta_{13} \cdot x^{AxT} - \kappa_{13} \cdot \sum_k \delta(t - t^L_k) \cdot x^{AxT}$<br>$[NT]^{OP} = \overline{[NT]}_{max} \cdot x^{AxT}$	Differential DNP **Eq.**(1)	

S3 Fig in S1 Appendix. Schematics of the multi-channel AL circuit with Pre-LN global feedback inhibition and the associated DNP circuit. (Left) Example multi-channel AL circuit with Pre-LN global feedback pathways and (Right) the associated DNP circuit. The multi-channel AL circuit has R channels, receiving input from OSNs expressing R receptor types. Shown above are channels 1 and R. The Pre-LN pathway receives as input the neurotransmitter concentration of OSN Axon-Terminals across channels and outputs a single Pre-LN spike train. As opposed to the cross-channel integration of the Pre-LN pathway, the Post-eLN and Post-iLN pathways are repeated for each channel and only perform computation local to the corresponding glomerulus. The same distinction of global vs. local computation is shown for the equivalent differential DNP circuit on the right. Here the multi-channel Pre-LN pathway is modeled as a *Global* DNP-FB. Refer to **S4 Table in S1 Appendix** for model details.

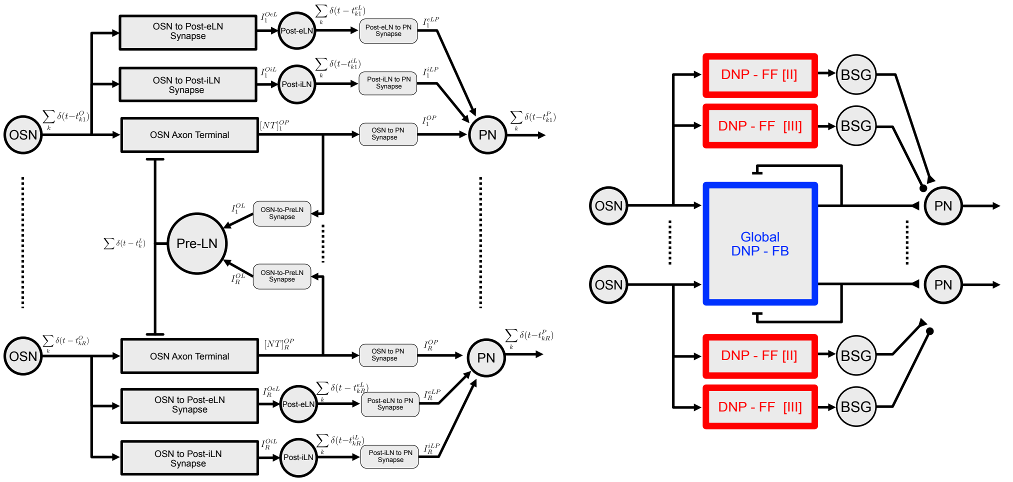

S4 Table in S1 Appendix. Model description of the multi-channel AL circuit in S3 Fig in S1 Appendix. Note that for notational simplicity the parameter values for $\boldsymbol{\theta}$ are omitted. Indices under the *Model* column refer to the following detailed models: (1) OSN to PreLN Synapse in the r-th channel, (2) Pre-LN BSG, (3) OSN Axon-Terminal in the r-th channel. Note that only components in the Global DNP-FB pathway are specified as the other circuit components are the same as in **Fig 11** (Left) for each of the R channels. The differential equations for each model are detailed under the *Equation* column, with the corresponding parameters summarized under the *Parameters* column. Note that under the *Parameters* column, values for the fixed parameters are shown. The circuit has a total of 23 free parameters, the same as the single-channel AL circuit in **S2 Fig in S1 Appendix** and **S3 Table in S1 Appendix**. The correspondence between the Pre-LN pathway and the Global DNP-FB model is made explicit on right side of rows (1-3).

Model	*Parameter*	*Equation*	*Differential DNP Operators*	
(1)	$[\alpha_{16}, \beta_{16}, \overline{g}^{OL}_{max}]$	$\frac{d}{dt}x^{OL}_r = \alpha_{16} \cdot [NT]^{OP}_r \cdot (1 - x^{OL}_r) - \beta_{16} \cdot x^{OL}_r$ $I^{OL}_r = \overline{g}^{OL}_{max} \cdot x^{OL}_r \cdot (V^L - E^{OL})$	$\mathcal{T}_5 : \sum_k \delta(t - t_{kr}) \mapsto \sum_k \delta(t - t_{kr})$	
(2)	$\sigma^L = 0$	$\sum_k \delta(t - t^L_k) \leftarrow \text{NoisyConnorStevens}(\sum_{r=1}^R I^{OL}_r; \sigma^L)$	$\mathcal{T}_6 : \{[NT]^{OP}_r\}_{r=1}^R \mapsto \sum_k \delta(t - t^L_k)$	Global DNP-FB
(3)	$[\alpha_{17}, \beta_{17}, \kappa_{17}, \overline{[NT]}_{max}]$	$\frac{d}{dt}x^{AxT}_r = \alpha_{17} \cdot \sum_k \delta(t - t^O_{kr}) \cdot (1 - x^{AxT}_r) - \beta_{17} \cdot x^{AxT}_r - \kappa_{17} \cdot \sum_k \delta(t - t^L_k) \cdot x^{AxT}_r$ $[NT]^{OP}_r = \overline{[NT]}_{max} \cdot x^{AxT}_r$	Differential DNP **Eq.**(4)	

S4 Fig in S1 Appendix. Robust ON/OFF detection for complex odorant concentration waveforms. (A [i]) Acetone concentration waveform with multiplicative white noise. (A [ii, ii]) Post-eLN/Post-iLN to PN synaptic current. (B) Same as in (A) for a Brownian motion concentration waveform. (C) Same as in (A) for step, ramp, and parabola concentration waveforms as in [1].

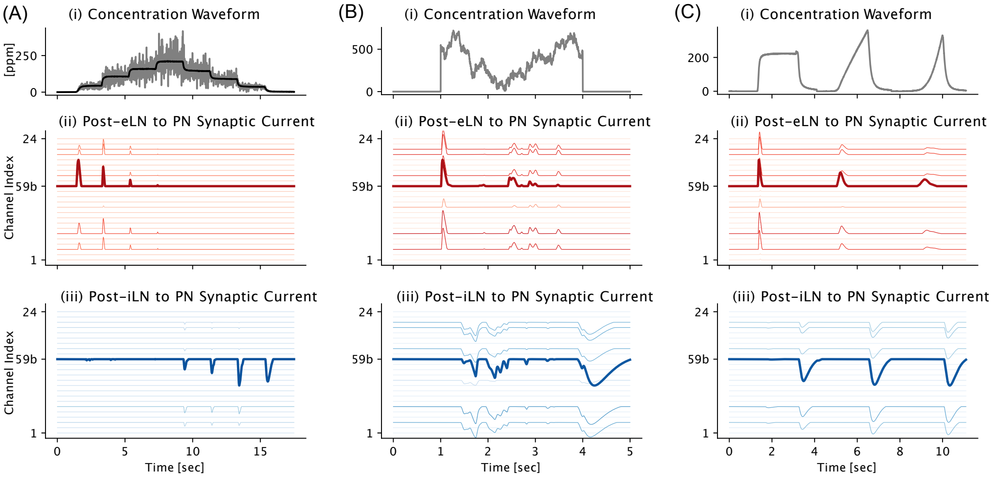

S5 Fig in S1 Appendix. Stability of Steady-State Or59b OSN and DM4 PN PSTHs. (A) Or59b OSN and DM4 PN PSTHs are shown in grey. The steady-state response component is, respectively, shown in red. (B) Coefficient of variation of the amplitudes of the piecewise constant response components (in red) of Or59b OSN and DM4 PN PSTHs. For each PSTH in (A), the amplitude of the piecewise constant response components after each jump time is recorded (e.g., 8 such values in (A top-left)). The resulting coefficient of variation (C.V.) for a given experiment is shown by the red triangles in (B). The box-plots describe the distribution of the C.V. of Or59b OSN and DM4 PN steady-state response amplitudes. Note that since the identity of the stable attractor limit cycle is directly related to the amplitude of the steady-state responses, the C.V. of the steady-state response amplitudes serves as a proxy for the stability of the stable attractor limit cycles. We observe that the C.V. of the DM4 PN responses has much lower values than the ones of the OSN responses.

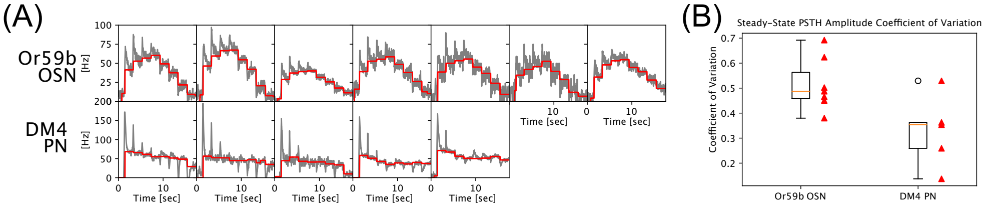

References

1. Lazar AA, Yeh CH. A molecular odorant transduction model and the complexity of spatio-temporal encoding in the Drosophila antenna. PLOS Computational Biology. 2020;16(4):e1007751. doi:10.1371/journal.pcbi.1007751.

2. Münch D, Galizia CG. DoOR 2.0 - Comprehensive Mapping of Drosophila melanogaster Odorant Responses. Scientific Reports. 2016;6(October 2015):1–14. doi:10.1038/srep21841.
