# Supplementary material for: The functional logic of odor information processing in the Drosophila antennal lobe
Source: PLoS Comput Biol. 2023 Apr 21;19(4):e1011043. doi: 10.1371/journal.pcbi.1011043 (PMC10156017; doi:10.1371/journal.pcbi.1011043)
Supplement: Attachment — Submitted filename: flyAntennalLobe_review.pdf [file pcbi.1011043.s002.pdf]

Summary

The *Drosophila* Antennal lobe (AL) is the first relay center for olfactory information processing. While the responses properties of the output of AL, the projection neurons (PNs) have been extensively studied, the functional logic of AL neural circuit in olfactory information processing is not fully understood. Various different type of Local neurons (LN) in AL have been thought to play important role in shaping the output of PNs. Based on previous experiment, in this paper, Lazar *et al* hypothesized that neural circuit in AL can segregate odorant identity and robustly detect odorant onset and offset. To test their hypothesis, they set out to explore all the possible single glomerulus models and multi-glomeruli models with different local neurons innervation patterns that enable PNs to have the above two properties. They found that presynaptic inhibition across glomeruli is essential to enhancing concentration-invariance of the PN responses, while postsynaptic LN excitation and inhibition strongly boost the responses of PNs to odorant onset and offset, respectively. Finally, the authors showed that the AL circuit is functionally equivalent to three parallel differential divisive normalization processors that extract the concentration-invariant and ON/OFF contrast-boosting features independently.

Given the highly conserved organization of olfactory systems across different species, understanding the computational function of AL have a much broader implication in early sensory systems. The authors approached this problem by extensive modeling of possible neural circuit in AL, providing an useful source for comparing the anatomy AL in different insects. The conclusion seems to be well supported by the numeric simulations. I would like to see the paper to be published in PLoS Computational Biology after the authors address the following issues

Major

The motivation of modeling framework

1) The authors hypothesized that the steady state PN responses are concentration-invariant which contains odorant identity information and the transient responses boost the detection of odorant onset and offset. This is based on experimental observation on a single channel/glomeurlus, Or59b/DM4, do other PNs have similar properties? The authors assume that " representation by the Projection Neurons (PNs) must recover odorant identity unambiguously in the face of concentration fluctuations" (Line 9-11). It is not clear whether or not odorant identity should be explicitly decoded at this stage. It is likely that odorant identity is decoded at the higher centers like lateral horn. The authors should provide experimental evidence to support this assumption.

2) The connectome for both fly larvae and adult AL are already available. Hence, the innervation patterns of LNs in AL is largely known. Why do the authors search all the possible circuits instead of focusing on known neural circuit in AL? A rationale in the introduction would be helpful.

3) The use of spiking model. There is always a trade-off between better fitting of data and interpretability. Complex models generally have better fitting of experimental data, but often lack transparency or intuition to understand. For example, in this study, to what extend are spiking models necessary to explain the two features of PNs: concentration invariance and ON/OFF detection? Since the comparison with experiment is all about instantaneous firing rate. Could simpler rate model be good enough? The authors should explain why they choose spiking model over rate model.

The model description is not clear

4) As a computational work, I expect that the modeling part contains enough details. For example, the authors stated "we modeled the Pre-LN inhibition of the OSN Axon-Terminal similar to the inhibition exerted by the calcium channel of the OTP". Without referring the cited reference 2, it is difficult for the readers to understand the difference between pre-iLNs and post-iLNs. Also, the meaning of each term in Eq. 1-4 is not easy to interpret. It would be much better if the authors can explain each term in model 1 in figure 9B. Or the authors could make an illustration to explain the "calcium channel of the OTP"?

Although the authors searched a very large parameter space, how did they choose the range of each parameter? It should be explained in the Material and Methods.

Objective function for odorant identity

5) In line 226-229, the authors stated "As the odorant identity is represented by the affinity vector in steady-state, the objective is the angular distance between the PN steady-state response and the odorant affinity vector". For me, this is not obvious and why would this be a good odorant identity objective function? For example, a more natural way to define the objective function is to train a simple decoder based on the responses patterns of PNs. The decoding error can be used as an objective function.

The model prediction are not clear

6) It would be great to see testable predictions from this modeling study. For example, the authors can link their three parallel differential DNP with known anatomical structure of AL. The authors can also make some comments on their model with previous models regarding biological adaptation. For example, in bacterial chemotaxis, both concentration-invariant response

and ON/OFF response are observed. The signaling transduction pathway and biophysical mechanisms are well understood.

Minor

1) The use of "temporal model" and "spatio-temporal" model are unconventional and might be confusing. "Temporal" typically refers to the dynamics of certain systems, while "spatio-temporal" are typically used to emphasize the spatial aspect of an changing signal, such as the spatial pattern of neural activities. I think in this study, they are used to refer "single channel/glomerulus" and "multi-channel" model. The authors can clarify this point.

2) As the authors aimed to exhaustively explore possible AL LN circuits, three types of innervation patterns are considered: pre-LN, post-iLN and post-eLN. Could the author explain why they eliminate presynaptic excitatory LNs to the terminal of OSN axons? Is that because no experiment support the existence of such LNs?

3) The model is really about the temporal response properties of PN to single odorant. The identity of an odorant is defined by the binding and disassociation constants vector. In natural environment, an odor typically consists of many different chemical compounds, for example, the smell of "coffee". How to define the identities of natural odors in terms of binding/disassociation constants of individual odorant molecules? For natural odors, both the composition (individual odorants molecules) and their relative abundances (concentrations) affect the perception of odors. It seems to be you can not segregate identity from concentration of individual odorant molecules. Some discussion would be helpful.

4) The waveforms of odorants used in this paper are relatively simple, i.e., step, ramp, exponential. Odor plumes encountered by animals in the natural environment would be much more complex, what's the implication of concentration invariance and ON/OFF detection to such odors? For natural odor plumes, the responses of PNs probably cannot be decomposed as steady responses and transient responses.

5) SNR is extensively used as a metric to quantify the goodness of fitting. In the definition (line 604), what is the "clean" signal and what is the "noise" when we interpret figure 3B? Some explanation in the main text when referring to figure 3 would be great.

6) How do you estimate the instantaneous firing rate in the experiment? If the original recording are spike trains, the width of sliding window might affect the noise level in the firing rate. This may in turn affect the SNR value in the model.

7) Definition of transient states? The PSTH seems to be quite noisy, is that because of the short time window used to calculate PSTH? If so, does different length of time windows affect

the SNR comparison. For example, what is you pass the instantaneous firing rate into a low pass filter before decomposing the steady state and transient state. I suspect the SNR values for different model might change.

8) Fonts in some figures are too small and hardly visible when printed on a Letter paper. I would increase the fonts like figure 3A and figure 4A.

9) The conclusion that "the steady-state and transient response features are, respectively, decoupled by the presynaptic and postsynaptic LNs" is not supported by figure 3. It seems that most of the models give very high correlation between steady state PN responses and the concentration waveform.

10) If odor identify information is encoded in the steady state firing rate of PNs which typically requires several hundreds ms to reach, how do you reconcile the observation that flies can recognize an odor within 100 ms (work from Rachel Wilson lab)? Further more, for highly dynamic natural odor plumes, the responses of PNs may never reach steady states. In this scenario, how does the AL encode odor identity?
