# Supplementary material for: The functional logic of odor information processing in the Drosophila antennal lobe
Source: PLoS Comput Biol. 2023 Apr 21;19(4):e1011043. doi: 10.1371/journal.pcbi.1011043 (PMC10156017; doi:10.1371/journal.pcbi.1011043)
Supplement: Attachment — Submitted filename: answers reviewer.pdf [file pcbi.1011043.s004.pdf]

The Functional Logic of Odor Information Processing in the *Drosophila* Antennal Lobe

Aurel A. Lazar[1,*], Tingkai Liu[1] and Chung-Heng Yeh[1]

[1] *Department of Electrical Engineering, Columbia University, New York, NY 10027, USA*
[1] *The authors' names are listed in alphabetical order.*
[*] *Corresponding author. Email address: aurel@ee.columbia.edu*

November 28, 2022

1 Response to the reviewers

Based on the responses of the reviewer, we have made the following changes:

- *In response to the request by both reviewers of making the paper more readable,* we have substantially restructured the manuscript. *In our revision, we focused on the development and analyses of two AL circuit models: single-channel (single glomerulus) in* Section 2.1 *and multi-channel (across glomeruli) in* Section 2.2*, with figures 3,4 redrawn to reflect the new structure. The comparative analyses of different circuit architectures that were previously the emphasis of 2.1 and 2.2 have been reduced to only cover the Pre-LN pathway (in the Discussion and Methods section). Other more extensive and exhaustive comparisons only appear now in the previously released preprint on BioRXiv [1].*
- *In response to the reviewer's comment regarding the response of the Antennal Lobe model to more complex odorant waveforms, in particular the concern regarding the lack of 'Steady-state' when the odorant concentration is more complex than the staircase waveform considered, we provided a different perspective on the AL response for complex stimuli. Instead of analyzing the Peri-Stimulus Time Histogram computed from the spiking output of the model, we now focus on the* phase-space representation *of limit cycles and their significance to the information representation at the PN single and population level. Consequently, figures 6, 7, 8, 9 have been redrawn to reflect the phase-space perspective. We have also significantly expanded upon the discussion around the robustness of the AL model in Section 2.3 and in supplementary materials. We show that the proposed functional logic of the AL circuit is largely invariant to concentration changes even for highly complex concentration waveforms.*
- *We believe that in the revised manuscript the question of how semantic information, often associated with subjective perception, can be characterized, is much more accessible to a wider audience. Moreover, it becomes abundantly clear, that the separation between the semantic information and syntactic information can not be tackled by single*

channel neurophysiology recordings. This observation highlights the need for renewed emphasis for multichannel neurophysiology recordings and formal characterizations of multi-input multi-output neural circuits.

- *Both reviewers also mentioned connecting the AL model to the available connectome data of the adult fly. While we agree that this is an important direction, it adds significant additional complexity to the current paper. We refer the reviewers to our recently released manuscripts on BioRXiv [2, 3] that explore this and other research directions. Reviewer 1 also suggested that we consider odorant mixture stimuli, a subject that we have studied in a followup paper [4]. We believe that the highly complex nature of odorant mixtures, especially the* semantic *information of a mixture of odorants, warrants a dedicated manuscript.*

Reviewer 1

Summary

The Drosophila Antennal lobe (AL) is the first relay center for olfactory information processing. While the responses properties of the output of AL, the projection neurons (PNs) have been extensively studied, the functional logic of AL neural circuit in olfactory information processing is not fully understood. Various different type of Local neurons (LN) in AL have been thought to play important role in shaping the output of PNs. Based on previous experiment, in this paper, Lazar et al hypothesized that neural circuit in AL can segregate odorant identity and robustly detect odorant onset and offset. To test their hypothesis, they set out to explore all the possible single glomerulus models and multi-glomeruli models with different local neurons innervation patterns that enable PNs to have the above two properties. They found that presynaptic inhibition across glomeruli is essential to enhancing concentration-invariance of the PN responses, while postsynaptic LN excitation and inhibition strongly boost the responses of PNs to odorant onset and offset, respectively. Finally, the authors showed that the AL circuit is functionally equivalent to three parallel differential divisive normalization processors that extract the concentration-invariant and ON/OFF contrast-boosting features independently.

Given the highly conserved organization of olfactory systems across different species, understanding the computational function of AL have a much broader implication in early sensory systems. The authors approached this problem by extensive modeling of possible neural circuit in AL, providing an useful source for comparing the anatomy AL in different insects. The conclusion seems to be well supported by the numeric simulations. I would like to see the paper to be published in PLoS Computational Biology after the authors address the following issues

Major

The motivation of modeling framework

1. The authors hypothesized that the steady state PN responses are concentration-invariant which contains odorant identity information and the transient responses boost the de-

tection of odorant onset and offset. This is based on experimental observation on a single channel/glomeurlus, Or59b/DM4, do other PNs have similar properties? The authors assume that " representation by the Projection Neurons (PNs) must recover odorant identity unambiguously in the face of concentration fluctuations" (Line 9-11). It is not clear whether or not odorant identity should be explicitly decoded at this stage. It is likely that odorant identity is decoded at the higher centers like lateral horn. The authors should provide experimental evidence to support this assumption.

- *We thank the reviewer for pointing out the main hypothesis in our work regarding the recovery of odorant identity at the level of the Projection Neurons in the Antennal Lobe. We'd like to highlight that the decomposition of the PN response into semantic information and syntactic information, is observed in DM4 PNs of different animals (See S1 Fig in Supplementary Material). Furthermore, as mentioned by both the reviewer and in our manuscript, to validate our hypothesis that the PNs represent odorant object identity requires the simultaneous physiological recording of all PNs across glomeruli for different odorant object identities and under different concentration amplitudes. Moreover, using the framework of syntactic vs. semantic information, our model of odorant stimuli suggests that it is not possible to prove/disprove the recovery of semantic information from single-channel recording data - a fundamental constraint that we cannot overcome given the datasets currently available. From a computational and theoretical standpoint, we argue that the the lack of synaptic depth of the early olfactory system (the number of synapses traversed in the direction of odor information flow) supports our hypothesis that odorant identity* could *be already recovered at the PN level. The reviewer proposed an alternative hypothesis that odorant identity may be identified in the LH. However, we are not aware of experimental data that include a quantitative characterization of odorant semantics supporting this hypothesis. We fully agree that experimental evidence supporting our hypothesis would be an exciting future direction and hope that our work provides a theoretical framework for future experimental work when such experiments are possible.*

2. The connectome for both fly larvae and adult AL are already available. Hence, the innervation patterns of LNs in AL is largely known. Why do the authors search all the possible circuits instead of focusing on known neural circuit in AL? A rationale in the introduction would be helpful.

   - *We thank the reviewer for pointing out the lack of justification for our methodology. We are very much aware that the structural information of the fly connectome/synaptome might help. We have been and continue exploring this research directions, see [2, 3]. However, this requires more extensive investigations that are well beyond the limits of this manuscript. We note also, that we have reduced the discussion on the comparative analyses of different circuit architectures. Nonetheless, we believe comparative analyses allow us to pose counter-factual questions such as "what if the circuit has no pan-glomerular local neurons?" - a question that computational research is uniquely suited to address, and allows us to better understand what role each LN type plays in the AL circuit. We've provided*

additional justifications for comparative analyses in Section 4.4 of the Methods section.

3. The use of spiking model. There is always a trade-off between better fitting of data and interpretability. Complex models generally have better fitting of experimental data, but often lack transparency or intuition to understand. For example, in this study, to what extend are spiking models necessary to explain the two features of PNs: concentration invariance and ON/OFF detection? Since the comparison with experiment is all about instantaneous firing rate. Could simpler rate model be good enough? The authors should explain why they choose spiking model over rate model.

    - *We thank the reviewer for pointing out the trade-off between complexity and model interpretability. In our work, the spike generation process of the neurons in the AL are included for the purpose of biological realism, and the optimization procedures are performed by evaluating the synaptic currents injected into PNs and not directly the PN output spike trains. The novelty of our work is in part based on the notion 'jump times'. These times delimit, e.g., the beginning and the end of odorant stimuli. This requires timing precision that PSTHs are lacking, an argument strongly supported by formal point process theory. Formal methods of time encoding, e.g., show that even minor jittering of spike times introduces noise and reduces the faithfulness of the representation of syntactic information.*

The model description is not clear

1. As a computational work, I expect that the modeling part contains enough details. For example, the authors stated "we modeled the Pre-LN inhibition of the OSN Axon-Terminal similar to the inhibition exerted by the calcium channel of the OTP". Without referring the cited reference 2, it is difficult for the readers to understand the difference between pre-iLNs and post-iLNs. Also, the meaning of each term in Eq. 1-4 is not easy to interpret. It would be much better if the authors can explain each term in model 1 in figure 9B. Or the authors could make an illustration to explain the "calcium channel of the OTP"? Although the authors searched a very large parameter space, how did they choose the range of each parameter? It should be explained in the Material and Methods.

    - *We thank the reviewer for pointing out the lack of clarity of the model description in our manuscript. We have significantly revised the presentation and expanded upon model description of the single-channel and multi-channel AL circuit. In the Materials and Methods section, we have provided detailed model description, along with associated free parameters, for two example circuits. Additionally, the relationship between Eq 1-4 and the AL circuit are also made explicit for the examples circuits.*

    - *The explanation regarding the choice of range for parameter values was indeed missing in the manuscript. We have included a paragraph describing the heuristics used to specified the parameter range in the Methods section on L734-741 in the optimization related portion of the Methods section.*

Objective function for odorant identity

1. In line 226-229, the authors stated "As the odorant identity is represented by the affinity vector in steady-state, the objective is the angular distance between the PN steady-state response and the odorant affinity vector". For me, this is not obvious and why would this be a good odorant identity objective function? For example, a more natural way to define the objective function is to train a simple decoder based on the responses patterns of PNs. The decoding error can be used as an objective function.

   - *We thank the reviewer for providing decoder as alternative to evaluating PN recovery of odorant identity vector. However, training an additional decoder to evaluate our model performance has the following problems: 1) it introduces additional variables into the evaluation of our model, 2) a different decoder will need to be trained for every circuit architecture and parameter set, rendering it computationally intractable. In contrast, the angular distance is a scale-invariant measure of similarity that requires no training, and does not introduce additional unknowns into the evaluation procedure. More importantly, we believe that for a model to achieve a low angular distance is a much stronger requirement than for it to achieve a low decoding error, since a low angular distance necessarily implies that a naive decoder based on such distance metric will result in low decoding error. We also note that angular distance requires far fewer assumptions about the choice of decoder.*

The model prediction are not clear

1. It would be great to see testable predictions from this modeling study. For example, the authors can link their three parallel differential DNP with known anatomical structure of AL. The authors can also make some comments on their model with previous models regarding biological adaptation. For example, in bacterial chemotaxis, both concentration-invariant response and ON/OFF response are observed. The signaling transduction pathway and biophysical mechanisms are well understood.

   - *We thank the reviewer for providing this feedback. We have included references to the literature [5] regarding how the DNPs abstract anatomical structure. Note however, that the point of view introduced here on semantic/syntactic information and making parallel with the existing literature is a major task. As far as we are aware of, there is no notion of semantics or of a quantitative characterization of semantic information for that matter in biological adaptation and bacterial chemotaxis.*

Minor

1. The use of 'temporal model', and 'spatio-temporal model' are unconventional and might be confusing. Temporal, typically refers to the dynamics of certain systems, while, spatio-temporal, are typically used to emphasize the spatial aspect of an changing signal, such as the spatial pattern of neural activities. I think in this study, they are used to refer, single-channel/glomerulus, and 'multi-channel' model. The authors can

clarify this point.

- *Thank you for the suggestion. To avoid confusion, we have changed the language used in the manuscript to "single channel" and "multi-channel".*

2. As the authors aimed to exhaustively explore possible AL LN circuits, three types of innervation patterns are considered: pre-LN, post-iLN and post-eLN. Could the author explain why they eliminate presynaptic excitatory LNs to the terminal of OSN axons? Is that because no experiment support the existence of such LNs?

   - *The LNs considered in the current AL circuit are indeed a subset of all of LN types in the fly Antennal Lobe. We took advantage of the known diversity in connectivity and neurotransmitter profiles of the LNs, and found that the 3 LN types considered in the current study are sufficient to explain the observed AL dynamics. We have added additional clarifications in L56-60 in the Introduction section, as well as in L458-465 in the Discussion section.*

3. The model is really about the temporal response properties of PN to single odorant. The identity of an odorant is defined by the binding and disassociation constants vector. In natural environment, an odor typically consists of many different chemical compounds, for example, the smell of "coffee". How to define the identities of natural odors in terms of binding/disassociation constants of individual odorant molecules? For natural odors, both the composition (individual odorants molecules) and their relative abundances (concentrations) affect the perception of odors. It seems to be you can not segregate identity from concentration of individual odorant molecules. Some discussion would be helpful.

   - *We thank the reviewer for providing this feedback. Since the physiological recording data motivating our discussion is for mono-molecular odorant only, we thought it appropriate to focus on this type of sensory modality in the current work. The implications of odorant mixtures on the AL circuit is a very interesting and highly complex topic, and we would like to refer the reviewer to our follow up study on bioRXiv[4] that discusses odorant mixture processing in both the Antennal Lobe and the Mushroom Body.*

4. The waveforms of odorants used in this paper are relatively simple, i.e., step, ramp, exponential. Odor plumes encountered by animals in the natural environment would be much more complex, what's the implication of concentration invariance and ON/OFF detection to such odors? For natural odor plumes, the responses of PNs probably cannot be decomposed as steady responses and transient responses.

   - *We have dedicated the Section 2.3 to discuss robustness of the AL processing to more complex odorant stimuli. We included additional experiments with more complex odorant waveforms such as staircase odorant waveform with additive white noise and nonstationary signals. In Figure 8 and Supplementary Figure 5, we show that both the odorant identity and on/off timing information are robustly processed by the AL circuit model. We hope that our results make it clear that the information about odorants and reasoning about their presence is primarily in*

the semantic and not the syntactic domain. Regarding the waveform complexity of odorant stimuli, we know from precise measurements that the odorant transduction process taking place in the sensila and OSN cilia acts as a low pass filter (LPF) with bandwidth of some 30 Hz [6]. Thus, experimenting with white noise stimuli only guarantees that the frequency spectrum the LPF is fully explored. White noise odorant stimuli bandlimited to some 30Hz have the same effect.

5. SNR is extensively used as a metric to quantify the goodness of fitti ng. In the definition (line 604), what is the "clean" signal and what is the "noise" when we interpret figure 3B? Some explanation in the main text when referring to figure 3 would be great.

   - *We've re-written the SNR definition on L623-624. As written, the 'Signal' is the physiology recording and the 'Noise' is the difference between the physiology recording and the model response.*

6. How do you estimate the instantaneous firing rate in the experiment? If the original recording are spike trains, the width of sliding window might affect the noise level in the firing rate. This may in turn affect the SNR value in the model.

   - *Following [7], the PSTH is estimated by a sliding window of duration 200 ms with 100 ms overlap for all models, which has been clarified in the Methods Section 4.2. Additionally, the PN responses are now analyzed in the phase-space of the PN BSGs, and we no longer rely on SNR as a metric of comparison. As such, the exact value of the SNR and its dependency on the PSTH computation is no longer significant. Instead, the comparison between model output and physiology recording can be visually observed directly in, e.g., Fig 3(B).*

7. Definition of transient states? The PSTH seems to be quite noisy, is that because of the short time window used to calculate PSTH? If so, does different length of time windows affect the SNR comparison. For example, what is you pass the instantaneous firing rate into a low pass filter before decomposing the steady state and transient state. I suspect the SNR values for different model might change.

   - *We've expanded upon the procedure with which the transient signal components are computed in the Methods section. The PSTH of the physiology recording is low-pass-filered at 50 Hz [7], with a sliding window of 200ms as noted above. We removed the comparative analyses of different AL circuit architectures that focused on using SNR values as a metric of comparison. Additionally, we clarified that the transient 'state' corresponds to traversal along the limit cycle attractors in the PN BSG's phase-space in response to on/off odorant timing information.*

8. Fonts in some figures are too small and hardly visible when printed on a Letter paper. I would increase the fonts like figure 3A and figure 4A.

   - *Figures 3 and 4 have been removed in the latest revision. We believe that this is not the case with the newly drawn figures.*

9. The conclusion that "the steady-state and transient response features are, respectively, decoupled by the presynaptic and postsynaptic LNs" is not supported by figure 3. It

seems that most of the models give very high correlation between steady state PN responses and the concentration waveform.

- *Figure 3 has been removed. Instead of focusing on steady-state vs transient, the comparative analysis in Methods section 4.4 and Figure 13 focuses on recovery of odorant identity in the pre-LN pathway, where the effect of different circuit architectures on odor semantic information recovery is clearly visible. We note that, the steady-state for real-time odorant processing language construct has been changed to stable attractors in the phase-space of PN Biophysical Spike Generators (as depicted in figure 6,7,8,9). In the revised manuscript, the correlation metric is no longer used.*

10. If odor identify information is encoded in the steady state firing rate of PNs which typically requires several hundreds ms to reach, how do you reconcile the observation that flies can recognize an odor within 100 ms (work from Rachel Wilson lab)? Further more, for highly dynamic natural odor plumes, the responses of PNs may never reach steady states. In this scenario, how does the AL encode odor identity?

- *Thank you for your question - does the work that you are mentioning refer to detecting the presence of an odorant concentration waveform or the recognition of its identity? The latter seems a bit difficult to ascertain as it may involve memory. In any event, the transient responses of the AL circuit that encode on/off timing information occur at a much faster time scale then the identity-encoding response in our experiments. Such transient responses would signal changes in odorant identy, providing downstream circuits with the information needed for further processing. We also note that while the current work proposes that the identity can be recovered at the output of the AL, it does not make any prediction about the exact recognition mechanism. The latter may involve, e.g., a form of predictive processing that could accelerate odorant recognition. In our work, we envisioned that the confounding representation of odorant semantic information (identity) and syntactic information (concentration) are disentangled at the PN level, clearly visible in the phase-space of PN BSGs. In the PN BSG phase-space, the identity information is mapped into stable limit cycle attractors whereas the syntactic information contained in the jump times is mapped into temporary traversals cross limit cycles to-and-from the stable attractors. Moreover in section 2.3, we show that even for highly dynamic odorant stimuli, this perspective on the odorant semantic information representation still holds true.*

Reviewer 2

Overview

Lazar and colleagues attempt to present the functional logic of a popular neural system, first-to-second-order olfactory processing. They tackle the D. melanogaster antennal lobe. This is a topical and timely investigation because wet-lab investigation has already elucidated the circuit role of many neurons in the antennal lobe (Wilson 2013), and connectomic work has revealed the logic of its wiring diagram (nearly) in its entirety (Schlegel et al. 2020). The

authors are interested in showing how the AL may dissociate odour identity ('semantic information') from odor concentration ('syntactic information'). They focus on three different roles local neurons (LNs) can play in the AL: LN-OSN inhibition, LN-PN inhibition and LN-PN excitation and describe their action as that of three differential Divisive Normalization Processors (DNPs).

The authors examine temporal coding in a single channel (DM4, section 2.1, critical for coding syntactic information) and temporal-spatial coding (two glomeruli, section 2,2, critical for coding identity). For 2.1, the authors build on their prior theoretical work (e.g. (Lazar and Yeh 2020)) and physiological work (Kim et al. 2015) to model the DM4 olfactory glomerulus (Or59b OSN) of the adult fly. They assume three specific classes of LNs (Pre-iLNs, Post-eLNs and Post-iLNs) and test 12 plausible configurations within DM4. With these simple configurations, each involving one OSN unit, one PN and one or two LNs, the authors simulated $\sim 5 \times 10^8$ circuit+parameterization combinations (up to 23 free parameters, randomly sampled), comparing to real DM4 physiological data to evaluate performance. In 2.2, the authors similarly assess 20 architectures. In agreement with other work (Olsen et al. 2010) they show that pre-synaptic global inhibition is necessary for odour identification.

In section 2.3, the authors seek an 'algorithmic' description for LN action in the AL. They compare it to a 'Divisive Normalization Processor', a concept the authors had previously built in their prior work on the fly visual system (Lazar et al. 2020). The authors reinterpret their architectures as composites of 'DNPs' in order to bring their work into a more normative framework. In section 2.4, they then asses whether the AL acts to signal the onset and offset of odor identities, something they term an 'ON-OFF odorant identity recovery processor'. Interestingly, they suggest that multi-neuron LN cell types may help increase the fidelity of odour identification, assuming each is differently parametrised and reducing the brain's optimization burden.

I commend the authors on making some python code available (`https://github.com/TK-21st/AntennalLobeLLY22`). The authors approach is considered, and their results are interesting to research community. I recommend their article for publication in PLOS. I do not necessitate revisions, but I have some thoughts on improvements that I would like the authors to consider, which I detail below. My comments mainly pertain to readability, comprehension and context. Lack of attention of these three points can make neuro- computational work less impactful upon the community than it might otherwise be, which is a problem I think. I say this as someone whose background is more in neurobiology and

neuroinformatics but little engineering familiarity - researchers of my fingerprint should be a large portion of the potential target audience, I feel.

Major

Connectome

I strongly think that, with the advent if a publicly available connectome (`https://neuprint.janelia.org/`, includes AL), neural work in D. melanogaster should now as a matter of course, try to relate their finding to what can be observed in the connectome (Schlegel et

al. 2020). There are at least 195 LNs in the AL, per hemisphere (Schlegel et al. 2020). These neurons have been broken down into 4 super classes based on gross morphology. These neurons vary in their degree of polarisation and connectivity patterns, and to a lesser degree their transmitter usage. Their identity matters. To this end, I strongly encourage the authors to attempt to contextualise their results within the connectome. Specifically, cell type candidates for their Pre-LNs, Post-eLNs and Post-iLNs relative to the DM4 glomerulus could be sought and found. Different LNs clearly have a bias to be pre- or post-synaptic to OSNs, but most do at least a bit of both, and there is variation between glomeruli. Providing the cell types helps enable future work by other authors to, for example, experimentally test the models from Lazar et al. by targeting specific cell types for experimentation (in particular for ideas in section 2.3 and 2.4). Specifically, the authors hypothesise that 3 DNPs 'function independently to capture concentration invariance, ON and OFF contrast boosting, respectively'. What is the substrate for this in the connectome? Are their proposed architectures discriminable from the connectome at some connectivity threshold, and at some level of neuron pooling? The authors are aware of and use these tools (`https://www.fruitflybrain.org/#/posts/resources`). Showing that a mechanistic connectome-accurate model for say, DM4, is equivalent to the more normative models in the paper, would be an interesting supplement.

- *We thank the reviewer for the suggestion. There is a deep question of methodology that the reviewer is raising, both directly and indirectly. Methodologically, the study of the functional logic of odor signal processing can be approached from a number of points of view including questions raised by thinking originating depending on ones background in system neuroscience, computational neuroscience, and theoretical neuroscience. We have approached this problem from all these different but often complementary points of view. The current manuscript focuses on studying the functional logic of odor signal processing from a computational/theory point of view. The 'aestetics' of computation/theory calls for abstractions that are inspired by but not necessarily coming out of experimental/systems neuroscience. In particular, the notion of semantics introduced here is based on extending classical information theory and it has not been raised by systems neuroscience. Of course, the connectome/synaptome datasets of the fly provide new ways to ask questions about the functional logic of odor signal processing. We are addressing some of these questions and avenues of research separately as they warrant substantial explorations. In this context, exploring the structure of adult fly connectome has been made publicly available at [3]. In the current work, we took advantage of the known diversity in connectivity and neurotransmitter profiles of the LNs, and found that the 3 LN types considered in the current study are sufficient to quantitatively explain the observed AL circuit dynamics. We have added additional clarifications in L56-60 in the Introduction section, as well as in L458-465 in the Discussion section.*

Writing

the paper is written in a dense manner. It's accessibility and range could be improved by using clearer, more simple language to more effectively communicate its science. The reader's understanding of certain principles is taken for granted. For example, in section 2.2 it is not immediately clear what is happening: the authors are now modelling a two-

channel circuit, where each channel has a different affinity for their simulated odor, acetone. Simple explanations like this are absent in a myriad of locations through the paper. This will unfortunately decrease the impact of the work at hand, not least because many interested in this area are pure biologists unfamiliar with terms taken from electrical engineering and commonly known terms in theoretical neuroscience. In particular, the concept of a DNP is critical to the paper but poorly explained within the main text of the paper. (A discussion of its use/implementation in the visual versus the olfactory system might also have been interesting to read)

- *We thank the reviewer for the suggestion and has made extensive revisions to the manuscript to improve the clarity of the writing. For a list of key changes in the revision to improve readability, please refer to the description at the top of this document. The substantial changes and restructuring of the presentation of our research results was in large part due to address this very issue raised by the reviewer.*
- *In the revised Section 2.1, we have also made explicit the connections and differences between the Differntial DNP model proposed in the current manuscript and the DNP model previously proposed for the fly visual system.*

Medium

1. If the models were given a short-hand name that helped remind readers what they contained, rather than given numerals, the text would be easier to follow. A (slightly long) version is seen in Fig.3.C inset.
    - *We have relegated the entirity of the comparative analyses to the Methods section 4.4 and supplementary materials. The revised main text deals only with the canonical single-channel and multi-channel AL circuits. We believe in the latest revision, the models are now clearly defined.*
2. The authors break LNs into three groups, "1) pre-synaptic pan-glomerular (innervating all glomeruli) inhibitory LNs (Pre-LNs), 2) post-synaptic uni-glomerular (innervating a single glomerulus) excitatory LNs (Post-eLNs), and 3) post-synaptic uni-glomerular inhibitory LNs (Post-iLNs)." When they introduce these groups, they do not immediately define what they are pre/post synaptic to. Later, they say 'presynaptic (to OSN-to-PN synapse)' and 'postsynaptic (to OSN-to-PN synapse)', but seeing as they are not discussing tri-partite synapses this is slightly confusing. Presynaptic here means LN-OSN, and postsynaptic means LN-PN.
    - *We thank the reviewer for the suggestion. We have added a sentence clarifying the location of the synapse that the LNs are innervating on L56-62. For theoretical and computational tractability, many simplifications of the AL connectomes have been introduced. To help clarify the terminology and improve readability, we have added two glossaries of terms and mathematical notations in the supplementary materials.*
3. Paragraph starting line 181, Post-eLN and Post-iLN are said to be *required* for contrast boosting. However, the addition of a Pe-LN also helps implement contrast

boosting?

- *We thank the reviewer for the suggestion. We removed in this revision the comparative analyses between AL circuit architectures with different Post-eLN/Post-iLN configurations. We note that by linearly decomposing the PN response into three components and optimizing the three LN pathways separately, the Pre-LN channel no longer produces a contrast boosting effect. We believe that, algorithmically, it is beneficial for the LN pathways to process syntactic (with the effect of contrast-boosting) information and semantic information independently. We look forward to future experiments that can test our hypothesis regarding the roles of the LNs in the AL circuit.*

4. In section 2.2, the authors seem to be modelling two parallel glomeruli. Each channel has a different binding affinity for the authors' simulated odor, acetone. It was not clear to me if one of the channels is an in silico DM4 and that is what is being compared to the in vivo DM4 PSTH in Fig4.B, and the other its foil. Or is the authors' measure for identity the correspondence across the affinity vector between both channels and the models output, seemingly Fig4.C? The main results text for this section could be made more understandable. Using simpler language is a strength.

   - *Section 2.2 has been completely restructured. Just to clarify, the circuit in Figure 4 (A1) in the previous version of the manuscript contained all channels (glomeruli) across the entire AL. We have also added an extensive description of the optimization procedure in Section 4.3 of the Methods section. Briefly, the affinity vector is estimated from physiological experiments [8, 9], and the result is considered to be the "ground-truth" representation of odorant identity. To measure the degree of the odorant identity recovery by our in silico model of the Antennal Lobe, the affinity vector is compared against the model PN response (in silico) .*

5. The Divisive Normalization Processor is something developed in the authors' previous work. The present work lacks a satisfactory qualitative explanation.

   - *We added additional clarification in the section 2.1 of the revised manuscript to clarify the DNP models. We would like to emphasize, however, that only the critical point solutions the Differential DNP model described in equation (1-5) relate directly to the DNP model previously proposed for the fly visual system [5] - which motivated us to describe the dynamical system models in the current manuscript as* differential *DNP model. This has been made explicit in L168-170. To provide more intuition, we also included on L167 a reference to the history of divisive normalization models [10].*

6. When the authors use the term 'PN' they always refer to the excitatory uniglomerular PNs. The fly contains many more multiglomerular PNs, whose function is less well understood. For clatity, I opine it is better to term these neurons uniglomerular PNs, i.e. uPNs, explicitly.

   - *The model proposed here is not a complete reflection of the AL connectome but rather a simplified model that captures the essential features of the AL computa-*

tion. We've added a discussion about the limitations of the model in the Discussion section around L458-466, and have also clarified the difference in nomenclature in glossary table in Supplementary Materials. For simplicity, we refer to uPNs as PNs, an abbreviation that is now mentioned in the glossary table in Supplementary Materials.

7. The existence of Pre-LNs, Post-eLNs and Post-iLNs are taken for granted. References to anatomical work establishing the existence of each, and a discussion of what is already known about their action is warranted. For example their roles in divisive normalisation and gain control. It is not clear to me why the potential of possible Pre-eLNs (the authors only consider Pre-iLNs, though they call them Pre-LNs) is ignored.

   - *We thank the reviewer for the feedback. The current manuscript focuses on studying the functional logic of the Antennal Lobe computation using a simplified model of the Antennal Lobe connectome. A study relating the model presented in the current work to the adult fly connectome is publicly available at [3]. In the current work, we took advantage of the known diversity in connectivity and neurotransmitter profiles of the LNs, and found that the 3 LN types considered in the current study are sufficient to explain the observed AL dynamics. We have added additional clarifications in L56-60 in the Introduction section, as well as in L458-465 in the Discussion section.*

8. The authors use their prior mode for the Calcium Feedback Loop of the Odorant Transduction Process (OTP). It and the choice to use it is not adequately qualtiatively explained in the main text, and so the reader easily misses out on some important framing.

   - *We provided more information on the modeling choice in Section 2.1 on L160-170.*

9. I think that making a paper's text as accessible as possible benefits every paper. A glossary of terms such as: Conor-Stevens point neuron, concentration-invariance, object identity recovery, contrast-boosting., semantic information, syntactic information, as well as anatomical terms used in the paper, would greatly assist non-specialist readers and increase the potential reader pool for the paper.

   - *We have added two glossary tables for both the terms and the mathematical notation used in the revised manuscript in the Supplementary materials section.*

10. The paper refers to an OSN Axon-hillock. I am not aware that insect neurons are understood to have an axon hillock in the manner established with mammalian neurons. I think this term might therefore be a little misleading, but am open to being corrected here by the authors.

    - *Indeed, Axon-Hillock is used only to help reference to the biological locality (spike initiation zone) of the spike generation process in insect neurons. We opted to remove all references to Axon-hillock, and simply refer to the Biophysical Spike Generator model of OSNs.*

11. The qualitative description of the models' free parameters in table 9.b could be greatly

improved.

- *We have added additional explanations in L164-L165 and in the table for the glossary of terms.*

Minor

1. Figure 3A, diagrams are small. Colouring neurons by their putative transmitter/sign and class would be helpful.
   - *Figure removed.*
2. The abbreviation PSTH (peristimulus time histogram) is not written out or explained anywhere, but the term is used frequently used.
   - *Addressed in the Methods section on L576-578.*
3. In abstract, 'chllenges' → 'challenges'
   - *Addressed*
4. Line 21, 'the any' → 'any'
   - *Addressed*
5. Very minor quibble, but 'fruit fly' is a misnomer for D. melanogaster. It is a vinegar fly. I guess this doesn't really matter but I though I'd throw it in there.
   - *We thank the reviewer for the quibble, but decided to follow the naming convention across the pond :)*
6. Figure 2, B - red shade in legend does not match plot.
   - *Figure 2 has been redrawn and the red shade is no longer part of the figure.*
7. Line 91, include citations.
   - *Introduction section has been re-written. All references are now included.*

References

1. Lazar, A. A., Liu, T. & Yeh, C.-H. The Functional Logic of Odor Information Processing in the Drosophila Antennal Lobe. *bioRxiv* (2022).
2. Lazar, A. A., Turkcan, M. K. & Zhou, Y. A Programmable Ontology Encompassing the Functional Logic of the Drosophila Brain. *Frontiers in Neuroinformatics* **16.** ISSN: 1662-5196 (2022).
3. Lazar, A. A., Turkcan, M. K. & Zhou, Y. A Programmable Model for Exploring the Functional Logic of the Drosophila Antennal Lobe. *bioRxiv* (2022).
4. Lazar, A. A., Liu, T., Yeh, C.-H. & Zhou, Y. Odorant Mixture Separation in Drosophila Early Olfactory System. *bioRxiv* (2022).

5. Lazar, A. A., Ukani, N. H. & Zhou, Y. Sparse identification of contrast gain control in the fruit fly photoreceptor and amacrine cell layer. *Journal of Mathematical Neuroscience* **10,** 3. ISSN: 21908567 (Dec. 2020).
6. Kim, A. J., Lazar, A. A. & Slutskiy, Y. B. System identification of Drosophila olfactory sensory neurons. *Journal of computational neuroscience* **30,** 143–161 (2011).
7. Kim, A. J., Lazar, A. A. & Slutskiy, Y. B. Projection neurons in Drosophila antennal lobes signal the acceleration of odor concentrations. *eLife,* e06651 (2015).
8. Lazar, A. A. & Yeh, C.-H. A molecular odorant transduction model and the complexity of spatio-temporal encoding in the Drosophila antenna. *PLOS Computational Biology* **16** (ed Louis, M.) e1007751. ISSN: 1553-7358 (Apr. 2020).
9. Münch, D. & Galizia, C. G. DoOR 2.0 - Comprehensive Mapping of Drosophila melanogaster Odorant Responses. *Scientific Reports* **6,** 1–14. ISSN: 20452322 (2016).
10. Carandini, M. & Heeger, D. J. Normalization as a canonical neural computation. *Nature Reviews Neuroscience* **13,** 51–62. ISSN: 1471-003X (Jan. 2012).
