# Supplementary material for: The functional logic of odor information processing in the Drosophila antennal lobe
Source: PLoS Comput Biol. 2023 Apr 21;19(4):e1011043. doi: 10.1371/journal.pcbi.1011043 (PMC10156017; doi:10.1371/journal.pcbi.1011043)
Supplement: Attachment — Submitted filename: flyAntennalLobe_review.pdf [file pcbi.1011043.s005.pdf]

The authors have addressed all my questions. The quality of the manuscript has largely improved. However, I have several minor questions to the authors before the manuscript is published.

1) In Figure 1B, the steady state response of DM4 PN is slightly higher for the gray waveform than that of black waveform. This is quit unexpected, the gray waveform has a lower concentration of odorant.

2) Please explain “phase-space” when it first appears in the main text. I think this will help readers to better understand the analysis.

3) The labels of axes of phase-space plots in figure 6 are missing.

4) The explanation of equation 1 is confusing. For example, $v(t)$ is the ratio of the ligand-bound receptor, what does “the co-receptor activation $x_1(t)$” mean? In *Drosophila*, the co-receptor of olfactory receptors is Orco. Only OR/Orco complex is functional.

5) In the abstract, the authors defined the syntactic information as the concentration. However, throughout the paper, syntactic information is more about the Onset/Offset of stimulus. In fact, the concentration information about each plateau of the stair waveform is totally lost at PN level.

6) Line 387. The authors claimed “the stable attractors remain largely unchanged”. It would be very helpful if they can quantify how stable they are. For example, Spearman’s rank correlation coefficient between two concentrations.

7) A discussion about how the AL circuit decode the absolute odorant concentration is necessary. Such information is presumably used by the olfactory systems. For example, an integration of a PN’s transient response against its concentration-invariant response may provide such information.
