# Supplementary material for: The functional logic of odor information processing in the Drosophila antennal lobe
Source: PLoS Comput Biol. 2023 Apr 21;19(4):e1011043. doi: 10.1371/journal.pcbi.1011043 (PMC10156017; doi:10.1371/journal.pcbi.1011043)
Supplement: Attachment — Submitted filename: Revised2 Answer to Reviewers.pdf [file pcbi.1011043.s006.pdf]

The Functional Logic of Odor Information Processing in the *Drosophila* Antennal Lobe

Aurel A. Lazar[1,*], Tingkai Liu[1] and Chung-Heng Yeh[1]

[1] *Department of Electrical Engineering, Columbia University, New York, NY 10027, USA*
[1] *The authors' names are listed in alphabetical order.*
[*] *Corresponding author. Email address: aurel@ee.columbia.edu*

February 27, 2023

1 Response to the reviewers

Overview of Changes

In this revision, we addressed all comments of the reviewers by again focusing on the clarity of our manuscript. While most changes were minor, we have made the following major changes:

- *Instead of equating ON/OFF responses of the Post-eLN/Post-iLN pathways as* syntactic information, *we now refer to them as* semantic timing *information. This change was made to address Reviewer 1's question regarding the exact definition of* syntactic information. *We emphasize that the* syntactic timing *information is defined as Shannon Information of the odorant concentration waveform at the sensory periphery, which influences (but does not equate) the ON/OFF timing information computed by the Post-eLN/Post-iLN pathways.*

Reviewer 1

1. In Figure 1B, the steady state response of DM4 PN is slightly higher for the gray waveform than that of black waveform. This is quit unexpected, the gray waveform has a lower concentration of odorant.

    - *After careful examination of the physiological recording data in* **Fig. 1** *and* **S1 Fig.**, *we concluded that while the observation of the reviewer is indeed correct, there is no clear monotonic relationship between the PN PSTH and the amplitudes of the staircase concentration waveform. As the exact PN PSTHs are subject to individual variability, we have decided to simplify* **Fig. 1** *and only show one of the experimental traces.*

2. Please explain "phase-space" when it first appears in the main text. I think this will help readers to better understand the analysis.

- *We thank the reviewer for the suggestion. A definition has been added to* **S1 Table.** *and a pointer was included on L80 of the revised manuscript where the term was first mentioned.*

3. The labels of axes of phase-space plots in figure 6 are missing.

   - *We thank the reviewer for pointing this out. We've included the axes limits and labels for the left-most 3d plots in Figure 6. (A, C). A note has been added to the caption stating that other 3d plots share the same axis limits and labels as the left-most plots. Additional labeling has been omitted to avoid clutter.*

4. The explanation of equation 1 is confusing. For example, $v(t)$ is the ratio of the ligand-bound receptor, what does "the co-receptor activation $x_1(t)$" mean? In Drosophila, the co-receptor of olfactory receptors is Orco. Only OR/Orco complex is functional.

   - We thank the reviewer for pointing this out. Following [1], x_1 is the gating variable of the co-receptor channel (addressed in line 168).

5. In the abstract, the authors defined the syntactic information as the concentration. However, throughout the paper, syntactic information is more about the Onset/Offset of stimulus. In fact, the concentration information about each plateau of the stair waveform is totally lost at PN level.

   - *We thank the reviewer for the feedback. We clarified in the latest revision that* syntactic *information is defined as the Shannon Information of the odorant concentration waveform, which influences, but does not equate, the ON/OFF timing information extracted by the AL circuit. Instead, we now refer to the ON/OFF timing information as* semantic timing. *See* **Overview of Changes** *at the beginning of this document for more details.*
   - *We clarify both here and on L76-78, L479-482, L604-605. of the revised manuscript that the concentration amplitude information is not lost, and is instead represented in the log-space as the temporal integral of the concentration contrast represented at the PN level.*

6. Line 387. The authors claimed "the stable attractors remain largely unchanged". It would be very helpful if they can quantify how stable they are. For example, Spearman's rank correlation coefficient between two concentrations.

   - *We thank the reviewer for the suggestion. To our knowledge, it has not been established how similarity between limit cycles in the phase-space can be formally quantified. We computed, however, the coefficient of variation (C.V., standard deviation divided by the mean) of the* amplitude *of the steady-state response components after each jump time as a proxy for quantifying the stability of the stable attractor limit cycles. The result in shown in a new supplementary figure* **S5 Fig.** *where we observe that the C.V. computed from DM4 PN response shows a much lower C.V. than that of the Or59b OSN.*

7. A discussion about how the AL circuit decode the absolute odorant concentration is necessary. Such information is presumably used by the olfactory systems. For example,

an integration of a PN's transient response against its concentration-invariant response may provide such information.

- *We thank the reviewer for the suggestion. We addressed this question on L76-78, L479-482, L604-605 of the revised manuscript. We note that the current work proposes that, for pure (mono-molecular) odorants, odorant identity can be readily decoded from the PN response in a concentration-invariant manner. Absolute concentration can in be principle obtained by integrating the contrast. Added in line 78.*

Reviewer 2

I thank Lazar and colleagues for their work in addressing my concerns and those of reviewer 1. In particular, in improving the readability of the manuscript. Having now read comments from reviewer 1, the authors' rebuttal and their revised manuscript I would like to re-hash one of my prior opinions rather strongly. I will not require that it needs be addressed for the paper to proceed - but I very strongly recommend to the authors that it should be, in service to the utility and impact of their work at a time for the field where linking mechanism and computation to identified neuronal hardware has never been easier and more fruitful.

I am not convinced by the authors' claim that they do not need to consider available connectome data in this work. Reviewer 1 suggested using the connectome very broadly, and the authors replied that this was not in scope. I agree with them on that front, but a simple connectome analysis should be done to at the very least contextualise their work. I raised this point strongly in my first review, and the authors essentially ignored it. In response to my points on connectome, anatomy, and prior literature, the authors did not answer my questions but told me that they are coming at the problem from a computational logic/theory point of view, and so use abstractions inspired by the systems neuroscience view but not necessarily linked to it. I understand this perfectly well and do not take issue with any of their abstractions - yet: there is no point in describing an abstract computation for the fly antennal lobe if the fly antennal lobe does not contain the wetware to run it. Indeed, figures 3 and 4 show model architectures that can be directly linked to the neurobiological network diagram - it is a small step!

The paper is about how the fly olfactory system - not something more abstract than that - and is based on anatomical connections observable within that system. The biological constraints are important. Very important, and the easier constraints to understand come from network structure - the authors have already tried hard in the more difficult case of neurophysiology. I am not asking the authors to use connectome-derived weights or exact neuron identities in their work, or build any kind of mechanistic model. Nor am I asking for a higher level characterisation of the network in terms of feedback loops and similar, as they present in another of their bioRxiv papers that they reference (rebuttal reference 3). Rather, it is totally in keeping with the nature of the work at hand to ask whether the motifs Lazar et al. are studying actually exist and in what proportions, and to what identified cell types they pertain - none of this is dealt with in reference 3. The latter point is instrumental in providing a starting point for experimentation that might validate some of this modelling

work. I am asking for a simple descriptive summary - in its simplest form, this might be some histograms or stacked bar plots showing the incidence of pre/post i/e LN-OSN connectivity. This is not about a clash of perspectives, it is about integration. To do otherwise at this time in the field is to knowingly limit the impact and usability of this work. To be clear again, I am not asking them to change anything about how their modelling is done. It is context I am after.

For example, does the connectome contain neurons that can be arranged as required for the authors' DNPs? How frequently do you find pre-LN, post-iLN and post-eLN innervation patterns, and does this differ between glomeruli? Do eLN-OSN inputs exist at all, or are they not important? Reviewer 1 also asked why the authors "eliminate presynaptic excitatory LNs to the terminal of OSN axons". I found the authors' reply, essentially that they did not need to, lacking. It is a clear and singular complement to the motifs they have already chosen. Its absence is conspicuous. Are there excitatory LN inputs to OSN axons in the fly brain? The authors say they "we believe that, algorithmically, it is beneficial for the LN pathways to process syntactic information and semantic information independently." Does this pathway separation anatomically exist?

The sum of my point here is: the authors put a lot of weight into trying different network configurations and showing us and explaining the model architecture and its operations but none into telling us - given the open source data available to them via Scheffer et al. and the annotations from Schlegel et al. - whether the fly has the neuronal architectures to support it.

We thank the reviewer for the detailed feedback. It appears to us that the reviewer's main concern is that the current work does not fully justify the proposed model architectures based on the available connectome data. We have clarified in the revised manuscript that mapping the Global Pre-LN to the connectome has been done in both larval [2] and adult AL [3, 4]. We have also clarified that excitatory postsynaptic LNs have also been reported in [5], while inhibitory postsynaptic LNs have been reported in larval AL [2] where uniglomerular PNs receive 20-40 percent synaptic input from GABAergic LNs. While we appreciate the reviewer's perspective - we thank the reviewer for suggesting the Hemibrain paper and dataset for which the reviewer was a co-author - we have to respectfully disagree with the reviewer's opinion that a connection to additional connectome data is necessary for the current work. Firstly, the reviewer seems to suggest that motivating the three LN types based on the previous literature is insufficient to justify "if the fly antennal lobe ... contain[s] the wetware to run it", and only a direct connection between the model architectures and the neurobiological network topology would suffice.

While we appreciate the reviewer's perspective, we hold a different assessment on the necessity of linking the model architectures to the neurobiological network topology within the scope of the current work. From our perspective, making a rigorous link between theoretical models and biological data require 3 components: 1) comparative connectomics analyses between multiple fly brains that provide conclusive evidence for the existence of certain motifs, 2) complete neurotransmitter profile information, 3) gap junctions information. We believe that single animal connectomes, while providing a wealth of information, are not sufficient to provide conclusive results describing LN motifs. This challenge is made particular apparent when

attempting to address reviewer's suggestion of describing "whether the motifs Lazar et al. are studying actually exist and in what proportions, and to what identified cell types they pertain"*, where we hesitate to draw any such conclusions based on single animal connectomics information as well as a lack of gap junction information in the AL.*

We strongly believe that the evidence provided by mapping the Global Pre-LN to the connectome in both larval [2] and adult AL [3, 4], the existence of excitatory postsynaptic LNs reported in [5] and inhibitory postsynaptic LNs reported in larval AL [2] where uniglomerular PNs receive 20-40 percent synaptic input from GABAergic LNs, are amply justifying the functional logic of the AL as a robust ON/OFF odorant object recovery processor. Nevertheless, we are open to be corrected on these points in the future by comparative connectomics, neurotransmitter profiles and gap junction datasets.

Data limitations aside, we believe the manuscript as it current stands remains sufficiently (perhaps overwhelmingly) dense in information as it provides, to our knowledge, the first hypothesis of how syntactic and semantic information are defined, measured and encoded in a biological system. A plethora of questions addressing the connection to biological data can be answered, some of which have been pointed out by the reviewer such as the separation of syntactic and semantic information in the AL. To address these questions, on top of the 37 pages current written, seems to be daunting for the potential readers.

More importantly, compared with the first version of the manuscript, we have significantly reduced the comparison between different AL circuit motifs for the exact reason of highlighting what we believe to be the most important contribution of the current work: an exact definition of "semantics" (odorant object identity) and semantic timing, (i.e., ON/OFF or arrival/departure of the odorant object) and how it may be processed by the Antennal Lobe. *From this perspective, connecting the circuit models to connectome data detracts the reader from the main message of the paper. Additionally, it seems to be a reasonable approach for us to present a circuit that accomplishes the hypothesized functional logic of the AL with the* minimal *biological wetware. We do not claim that the proposed circuit is the only possible realization that could achieve the same functional logic, but merely hypothesize based on what we know of the AL circuitry from [2–6]. In another word, we only claim that our model is sufficient to achieve the hypothesized functional logic, while the reviewer seems to suggest that we go one step further to explore whether it is "necessary" by considering other types of LNs (e.g., eLN-OSN).*

In summary, we have included additional clarifications in the revised manuscript to provide context of the choice of LN pathways considered in our model. We believe that connecting our model to connectome datasets is an incredibly valuable, but equally challenging task. We feel that we should not forget that, even under ideal conditions, these datasets have major limitations as they do not include, for example, gap junction information. To maintain the clarity of the paper, and the focus on its main message of functional logic, we have decided to leave this task for future works.

References

1. Lazar, A. A. & Yeh, C.-H. A molecular odorant transduction model and the complexity of spatio-temporal encoding in the Drosophila antenna. *PLOS Computational Biology* **16** (ed Louis, M.) e1007751. ISSN: 1553-7358 (2020).
2. Berck, M. E. *et al.* The wiring diagram of a glomerular olfactory system. *eLife* **5,** 1–21. ISSN: 2050-084X (2016).
3. Lazar, A. A., Turkcan, M. K. & Zhou, Y. A Programmable Ontology Encompassing the Functional Logic of the Drosophila Brain. *Frontiers in Neuroinformatics* **16.** ISSN: 1662-5196 (2022).
4. Lazar, A. A., Turkcan, M. K. & Zhou, Y. A Programmable Model for Exploring the Functional Logic of the Drosophila Antennal Lobe. *bioRxiv* (2022).
5. Tootoonian, S. & Laurent, G. Electric Times in Olfaction. *Neuron* **67,** 903–905. ISSN: 0896-6273. https://www.sciencedirect.com/science/article/pii/S0896627310007282 (2010).
6. Scheffer, L. K. *et al.* A connectome and analysis of the adult *Drosophila* central brain. *eLife* **9,** e57443 (2020).
