# Supplementary material for: The functional logic of odor information processing in the Drosophila antennal lobe
Source: PLoS Comput Biol. 2023 Apr 21;19(4):e1011043. doi: 10.1371/journal.pcbi.1011043 (PMC10156017; doi:10.1371/journal.pcbi.1011043)
Supplement: Attachment — Submitted filename: plos_comp_#1.docx [file pcbi.1011043.s003.docx]

## Overview

Lazar and colleagues attempt to present the functional logic of a popular neural system, first-to-second-order olfactory processing. They tackle the *D. melanogaster* antennal lobe. This is a topical and timely investigation because wet-lab investigation has already elucidated the circuit role of many neurons in the antennal lobe [(Wilson 2013)](https://paperpile.com/c/uASppC/SsMD), and connectomic work has revealed the logic of its wiring diagram (nearly) in its entirety [(Schlegel et al. 2020)](https://paperpile.com/c/uASppC/eYvX). The authors are interested in showing how the AL may dissociate odour identity (‘semantic information’) from odor concentration (‘syntactic information’). They focus on three different roles local neurons (LNs) can play in the AL: LN-OSN inhibition, LN-PN inhibition and LN-PN excitation and describe their action as that of three differential Divisive Normalization Processors (DNPs).

The authors examine temporal coding in a single channel (DM4, section 2.1, critical for coding syntactic information) and temporal-spatial coding (two glomeruli, section 2,2, critical for coding identity). For 2.1, the authors build on their prior theoretical work (e.g. [(Lazar and Yeh 2020)](https://paperpile.com/c/uASppC/eOpf)) and physiological work [(Kim et al. 2015)](https://paperpile.com/c/uASppC/8MLy) to model the DM4 olfactory glomerulus (Or59b OSN) of the adult fly. They assume three specific classes of LNs (Pre-iLNs, Post-eLNs and Post-iLNs) and test 12 plausible configurations within DM4. With these simple configurations, each involving one OSN unit, one PN and one or two LNs, the authors simulated ~5 × 10^8 circuit+parameterization combinations (up to 23 free parameters, randomly sampled), comparing to real DM4 physiological data to evaluate performance. In 2.2, the authors similarly assess 20 architectures. In agreement with other work [(Olsen et al. 2010)](https://paperpile.com/c/uASppC/ROA7) they show that pre-synaptic global inhibition is necessary for odour identification.

In section 2.3, the authors seek an ‘algorithmic’ description for LN action in the AL. They compare it to a ‘Divisive Normalization Processor’, a concept the authors had previously built in their prior work on the fly visual system [(Lazar et al. 2020)](https://paperpile.com/c/uASppC/gpr8). The authors reinterpret their architectures as composites of ‘DNPs’ in order to bring their work into a more normative framework. In section 2.4, they then asses whether the AL acts to signal the onset and offset of odor identities, something they term an ‘ON-OFF odorant identity recovery processor’. Interestingly, they suggest that multi-neuron LN cell types may help increase the fidelity of odour identification, assuming each is differently parametrised and reducing the brain’s optimization burden.

I commend the authors on making some python code available (<https://github.com/TK-21st/AntennalLobeLLY22>). The authors approach is considered, and their results are interesting to research community. I recommend their article for publication in PLOS.

I do not necessitate revisions, but I have some thoughts on improvements that I would like the authors to consider, which I detail below. My comments mainly pertain to readability, comprehension and context. Lack of attention of these three points can make neuro-computational work less impactful upon the community than it might otherwise be, which is a problem I think. I say this as someone whose background is more in neurobiology and neuroinformatics but little engineering familiarity - researchers of my fingerprint should be a large portion of the potential target audience, I feel.

## Major points

Connectome - I strongly think that, with the advent if a publicly available connectome (<https://neuprint.janelia.org/>, includes AL), neural work in *D. melanogaster* should now as a matter of course, try to relate their finding to what can be observed in the connectome [(Schlegel et al. 2020)](https://paperpile.com/c/uASppC/eYvX). There are at least 195 LNs in the AL, per hemisphere [(Schlegel et al. 2020)](https://paperpile.com/c/uASppC/eYvX). These neurons have been broken down into 4 super classes based on gross morphology. These neurons vary in their degree of polarisation and connectivity patterns, and to a lesser degree their transmitter usage. Their identity matters. To this end, I strongly encourage the authors to attempt to contextualise their results within the connectome. Specifically, cell type candidates for their Pre-LNs, Post-eLNs and Post-iLNs relative to the DM4 glomerulus could be sought and found. Different LNs clearly have a bias to be pre- or post-synaptic to OSNs, but most do at least a bit of both, and there is variation between glomeruli. Providing the cell types helps enable future work by other authors to, for example, experimentally test the models from Lazar et al. by targeting specific cell types for experimentation (in particular for ideas in section 2.3 and 2.4). Specifically, the authors hypothesise that 3 DNPs ‘function independently to capture concentration invariance, ON and OFF contrast boosting, respectively’. What is the substrate for this in the connectome? Are their proposed architectures discriminable from the connectome at some connectivity threshold, and at some level of neuron pooling? The authors are aware of and use these tools (<https://www.fruitflybrain.org/#/posts/resources>). Showing that a mechanistic connectome-accurate model for say, DM4, is equivalent to the more normative models in the paper, would be an interesting supplement.

Writing - the paper is written in a dense manner. It’s accessibility and range could be improved by using clearer, more simple language to more effectively communicate its science. The reader’s understanding of certain principles is taken for granted. For example, in section 2.2 it is not immediately clear what is happening: the authors are now modelling a two-channel circuit, where each channel has a different affinity for their simulated odor, acetone. Simple explanations like this are absent in a myriad of locations through the paper. This will unfortunately decrease the impact of the work at hand, not least because many interested in this area are pure biologists unfamiliar with terms taken from electrical engineering and commonly known terms in theoretical neuroscience. In particular, the concept of a DNP is critical to the paper but poorly explained within the main text of the paper. (A discussion of its use/implementation in the visual versus the olfactory system might also have been interesting to read)

## Medium points

If the models were given a short-hand name that helped remind readers what they contained, rather than given numerals, the text would be easier to follow. A (slightly long) version is seen in Fig.3.C inset.

The authors break LNs into three groups, “1) pre-synaptic pan-glomerular (innervating all glomeruli) inhibitory LNs (Pre-LNs), 2) post-synaptic uni-glomerular (innervating a single glomerulus) excitatory LNs (Post-eLNs), and 3) post-synaptic uni-glomerular inhibitory LNs (Post-iLNs).” When they introduce these groups, they do not immediately define what they are pre/post synaptic to. Later, they say ‘presynaptic (to OSN-to-PN synapse)’ and ‘postsynaptic (to OSN-to-PN synapse)’, but seeing as they are not discussing tri-partite synapses this is slightly confusing. Presynaptic here means LN-OSN, and postsynaptic means LN-PN.

Paragraph starting line 181, Post-eLN and Post-iLN are said to be *required* for contrast boosting. However, the addition of a Pe-LN also helps implement contrast boosting?

In section 2.2, the authors seem to be modelling two parallel glomeruli. Each channel has a different binding affinity for the authors’ simulated odor, acetone. It was not clear to me if one of the channels is an in silico DM4 and that is what is being compared to the in vivo DM4 PSTH in Fig4.B, and the other its foil. Or is the authors’ measure for identity the correspondence across the affinity vector between both channels and the models output, seemingly Fig4.C? The main results text for this section could be made more understandable. Using simpler language is a strength.

The Divisive Normalization Processor is something developed in the authors’ previous work. The present work lacks a satisfactory qualitative explanation.

When the authors use the term ‘PN’ they always refer to the excitatory uniglomerular PNs. The fly contains many more multiglomerular PNs, whose function is less well understood. For clatity, I opine it is better to term these neurons uniglomerular PNs, i.e. uPNs, explicitly.

The existence of Pre-LNs, Post-eLNs and Post-iLNs are taken for granted. References to anatomical work establishing the existence of each, and a discussion of what is already known about their action is warranted. For example their roles in divisive normalisation and gain control. It is not clear to me why the potential of possible Pre-eLNs (the authors only consider Pre-iLNs, though they call them Pre-LNs) is ignored.

The authors use their prior mode for the Calcium Feedback Loop of the Odorant Transduction Process (OTP). It and the choice to use it is not adequately qualtiatively explained in the main text, and so the reader easily misses out on some important framing.

I think that making a paper’s text as accessible as possible benefits every paper. A glossary of terms such as: Conor-Stevens point neuron, concentration-invariance, object identity recovery, contrast-boosting., semantic information, syntactic information, as well as anatomical terms used in the paper, would greatly assist non-specialist readers and increase the potential reader pool for the paper.

The paper refers to an OSN Axon-hillock. I am not aware that insect neurons are understood to have an axon hillock in the manner established with mammalian neurons. I think this term might therefore be a little misleading, but am open to being corrected here by the authors.

The qualitative description of the models’ free parameters in table 9.b could be greatly improved.

## Minor points

Figure 3A, diagrams are small. Colouring neurons by their putative transmitter/sign and class would be helpful.

The abbreviation PSTH (peristimulus time histogram) is not written out or explained anywhere, but the term is used frequently used.

In abstract, ‘chllenges’ -> ‘challenges’

Line 21, ‘the any’ -> ‘any’

Very minor quibble, but ‘fruit fly’ is a misnomer for D. melanogaster. It is a vinegar fly. I guess this doesn’t really matter but I though I’d throw it in there.

Figure 2, B - red shade in legend does not match plot.

Line 91, include citations.
